# Hypoglossal acupuncture for acute chemotherapy-induced dysgeusia in patients with breast cancer: study protocol of a randomized, sham-controlled trial

**DOI:** 10.1186/s13063-019-3525-y

**Published:** 2019-07-04

**Authors:** Heidemarie Haller, Taige Wang, Romy Lauche, Kyung-Eun Choi, Petra Voiß, Sabine Felber, Holger Cramer, Beyhan Ataseven, Sherko Kümmel, Anna Paul, Gustav Dobos

**Affiliations:** 1 Department of Internal and Integrative Medicine, Kliniken Essen-Mitte, Faculty of Medicine, University of Duisburg-Essen, Am Deimelsberg 34a, 45276 Essen, Germany; 2 Breast Unit, Kliniken Essen-Mitte, Essen, Germany; 3 Australian Research Centre in Complementary and Integrative Medicine (ARCCIM), Faculty of Health, University of Technology Sydney (UTS), Sydney, Australia; 4 Institute for Medical Sociology, Health Services Research, and Rehabilitation Science (IMVR) at the Faculty of Human Sciences and the Faculty of Medicine of the University of Cologne, Cologne, Germany; 5 Department of Gynecology and Gynecologic Oncology, Kliniken Essen-Mitte, Essen, Germany

**Keywords:** Acupuncture, Cancer, Chemotherapy, Dysgeusia, Taste disorders, Randomized controlled trial

## Abstract

**Background:**

Distortion of taste sensations is a common chemotherapy-induced side effect; however, treatment evidence is limited. Pilot data indicated that acupuncture might be able to improve symptoms of dysgeusia. Thus, the aim of this study is to investigate the effects and side effects of hypoglossal acupuncture in the treatment of dysgeusia in patients with breast cancer undergoing chemotherapy.

**Methods/design:**

The study is a randomized controlled trial comparing a single verum acupuncture treatment with two active comparators: sham acupuncture and dietary recommendations. Sample size calculation revealed a total of 75 patients pending an alpha of 0.05, a power of 0.8, and an estimated effect size of 0.80. Patients with breast cancer undergoing platinum- or taxane-based chemotherapy will be included if they present with phantogeusia (abnormal taste sensations without an external oral stimulus) with an intensity of 4 points or above on an 11-point numeric rating scale (NRS). The primary outcome is phantogeusia; secondary outcomes include parageusia (abnormal taste of food), hypogeusia (reduced taste sensations), hypergeusia (increased taste sensations), xerostomia (dry mouth), stomatitis, appetite, and functional impairment. All outcomes will be assessed at baseline and prior to the next chemotherapy administration using an 11-point NRS for each. All adverse events will be recorded.

**Discussion:**

The results of this study will demonstrate the extent to which hypoglossal acupuncture may influence the intensity of and functional impairment due to chemotherapy-induced dysgeusia.

**Trial registration:**

Clinical Trials.gov, NCT02304913. Registered on 19 November 2014.

## Background

Taste disorders are frequent side effects in patients treated with chemotherapy [1, 2]. According to the National Cancer Institute Common Terminology Criteria for Adverse Events, dysgeusia is defined as a disorder characterized by abnormal or impaired sense of taste with mostly unpleasant metallic, bitter, or salty alteration of taste sensations [3]. Subconditions of dysgeusia include phantogeusia, an abnormal sensation of taste without presence of an external oral stimulus; parageusia, an abnormal taste of food; hypogeusia, a reduced sensation of taste; and hypergeusia, an increased sensation of taste. Although most forms of dysgeusia present persistent courses in quite a few cases, prevalence during chemotherapy ranged between 50% and 75% [1, 4], which is shown to significantly affect patients’ quality of life and may cause malnutrition, weight loss, and increased morbidity [3, 5, 6].

Cytostatic and cytotoxic chemotherapeutics mainly induce dysgeusia by destroying olfactory and taste receptor structures due to their inherently high turnover rates of 7 to 10 days. Several antineoplastic agents themselves also have metallic- or bitter-tasting contents that can reach taste receptors by diffusion through capillaries [1]. Direct sensitization or damage of neurons, moreover, can alter afferent taste pathways without the need for destroyed receptor cells or bad-tasting molecules from chemotherapeutic drugs [3]. Other chemotherapy side effects, such as oral mucositis, gastroesophageal reflux, or infections, and the intake of antibiotics or analgesics used to manage these side effects can exacerbate existing taste disturbances [2, 7].

Evidence for effective pharmacological treatment strategies of chemotherapy-induced dysgeusia is limited. Although current studies do not support the intake of amifostine [8, 9] or the substitution with zinc [10] or glutamine [11], dietetic counseling has provided the best available evidence to date and is recommended by several systematic reviews [1–3, 12] and the American Institute for Cancer Research. The effects of acupuncture for the treatment of dysgeusia have been examined in only one pilot trial in 37 patients with idiopathic dysgeusia, showing significant benefits of selected body and ear acupuncture points over sham acupuncture [13]. A literature search of standard medical databases revealed no randomized controlled acupuncture trials for dysgeusia in patients undergoing chemotherapy [14], but evidence was found for several other chemotherapy-related symptoms, such as nausea and vomiting and leukopenia, and a lower quality of evidence for pain, hot flashes, fatigue, and xerostomia [15–17]. Because in Traditional Chinese Medicine, taste disorders and functional disturbances of the tongue were particularly treated with needling of hypoglossal acupuncture points [18, 19], the aim of the present study is to investigate the effects of acupuncture under the tongue for acute chemotherapy-induced dysgeusia.

## Methods/design

### Objectives

The aim of this study is to examine the efficacy and safety of hypoglossal acupuncture in the treatment of acute dysgeusia in patients with breast cancer undergoing chemotherapy.

### Ethics

The trial protocol has been approved by the ethics committee of the Medical Faculty of the University of Duisburg-Essen, Germany (approval number 14–5953-BO), is registered with ClinicalTrials.gov (NCT02304913), and will be conducted in accordance with the ethical principles for clinical trials as defined by the Declaration of Helsinki. Before inclusion in the study, all patients have to provide written informed consent.

### Study design

**Fig. 1:**
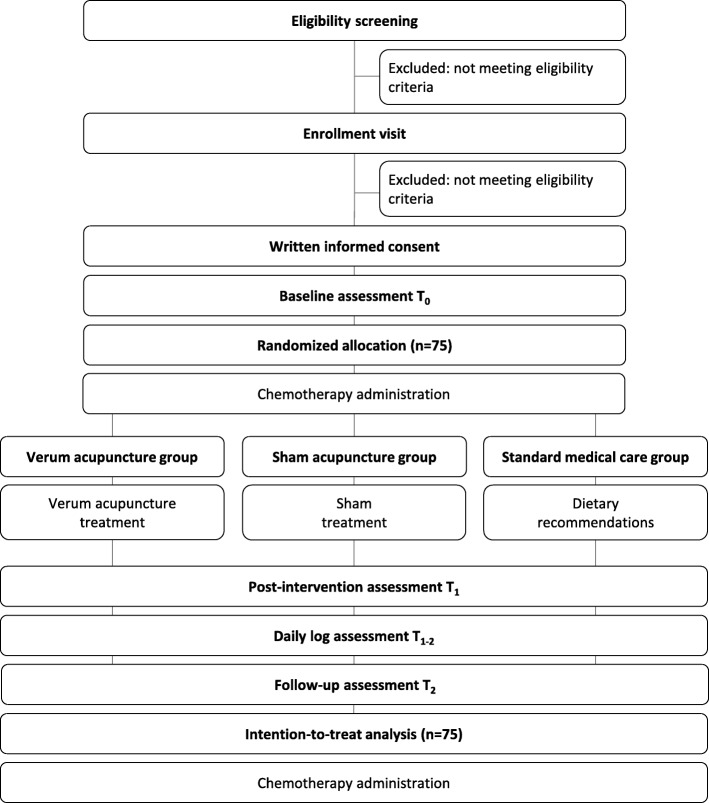
CONSORT flow chart

**Fig. 2:**
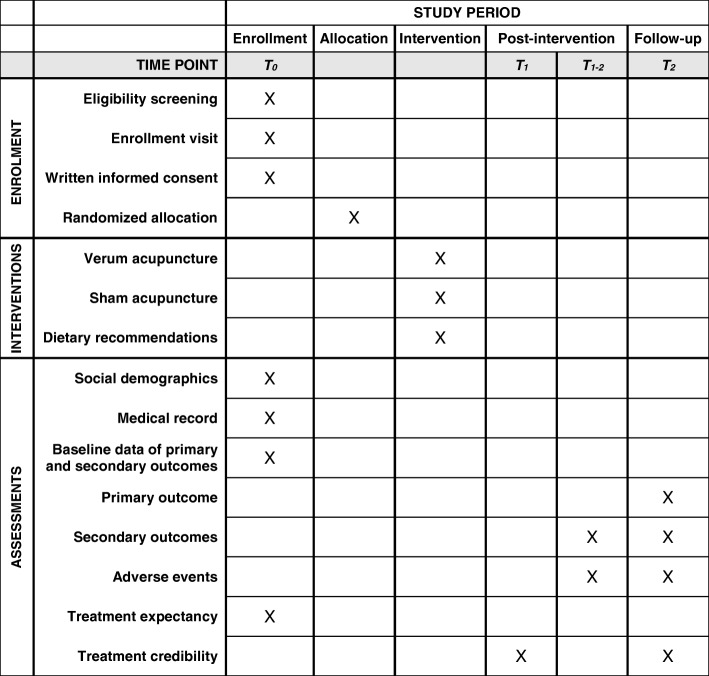
SPIRIT figure

The study is a randomized controlled clinical trial comparing verum acupuncture with two active comparators: sham acupuncture and standard medical care. It is designed according to the Standard Protocol Items: Recommendations for Interventional Trials (SPIRIT) 2013 statement [20], the Consolidated Standards of Reporting Trials (CONSORT) 2010 statement [21], and its Standards for Reporting Interventions in Clinical Trials of Acupuncture (STRICTA) 2010 extension [22]. If patients develop dysgeusia during chemotherapy and meet the eligibility criteria of this study, baseline data will be assessed at the individual’s next chemotherapy appointment, directly before chemotherapy administration (T_0_). Afterward, patients will be randomized 1:1:1 to one of the three groups. Patients of the acupuncture groups will receive either verum or sham acupuncture; patients of the standard medical care group will receive written, evidence-based dietary recommendations. Outcomes will be reassessed just before the next chemotherapy administration (T_2_) as well as between the chemotherapy administrations using a daily patient log (T_1–2_) (Figs. 1 and 2). Patient recruitment started in January 2015. Estimated primary completion date is December 2019.

### Randomization

A researcher who was not involved in conducting the study generated a stratified (by age and type of chemotherapy because both variables are found to affect the course of dysgeusia [23, 24]) random allocation sequence with randomly varying block lengths using Random Allocation Software [25]. The randomization list is password-secured, and no person other than the aforementioned researcher is able to access it. On the basis of this list, the researcher prepared sealed and opaque envelopes sorted in ascending order of randomization. Allocation to the groups will be executed by the study physician, who is involved in neither the random sequence generation nor the assessment of study outcomes.

### Blinding

First, patients of the two acupuncture groups will be blinded to whether they receive verum or sham acupuncture. Second, the outcome assessors delivering questionnaires to patients will remain blind to patients’ group allocations during the whole study period. Third, the statistician will be blinded to group allocation during data analysis by renaming groups with numbers.

### Participants

Patients will be recruited from the Department of Gynecology & Gynecologic Oncology, Kliniken Essen-Mitte, Germany. Interested patients with symptoms of phantogeusia are screened by the attending physician and receive detailed written study information and have an enrollment visit with the study physician on the day of their next chemotherapy administration. For inclusion in the study, all eligibility criteria have to be met and written informed consent must be obtained from the patient prior to inclusion.

Inclusion criteria are as follows:

- Patients with breast cancer (TNM stages I–III) undergoing initial treatment with platinum- or taxane-based chemotherapy (regardless of the length of the chemotherapy cycle)
- Phantogeusia intensity of 4 points or above on average on an 11-point numeric rating scale (NRS) since the last chemotherapy administration
- Willingness to participate in the study and provision of written informed consent

Exclusion criteria are as follows:

- Severe stomatitis
- Dysgeusia before chemotherapy (based on neurological diseases, diabetes, or ingestion of drugs with taste disorders as a side effect)
- Leukopenia/neutropenia
- Treatment with anticoagulants and/or hemophilia
- Smoker
- Severe physical or mental comorbidity (whereby the patient is unable to participate in the study)
- Use of other complementary treatments that might influence patients’ taste
- Participation in other studies of interventions for oral complications due to chemotherapy

### Interventions

#### Verum acupuncture group

Patients of the verum group receive a single treatment of hypoglossal needle acupuncture during their next chemotherapy administration after study inclusion. Treated acupuncture points will be Jinjin (Golden Liquid/EX-HN12) left beside the lingual frenulum and Yuye (Jade Fluid/EX-HN13) right beside the lingual frenulum. Both points are treated in quick succession with immediate removal of the needle (Table 1).

**Table 1:** Details of the acupuncture treatment according to STRICTA

STRICTA items		Detailed protocol description
Acupuncture rationale	(a) Style of acupuncture	Traditional Chinese needle acupuncture
	(b) Reasoning for treatment provided	Selected traditional acupuncture points for dysfunction of the tongue
	(c) Extent to which treatment was varied	Acupuncture with immediate removal of the needle
Details of needling	(a) Number of needle insertions	Two needle insertions per subject
	(b) Names of points used	Jinjin (Ex-HN 12), Yuye (Ex-HN13)
	(c) Depth of insertion	1–2 mm
	(d) Response sought	No De-Qi sensation
	(e) Needle stimulation	No stimulation
	(f) Needle retention time	2–3 s for each acupuncture point
	(g) Needle type	TeWa CB-Type 2540 (0,25 × 40 mm), medical stainless steel with copper handle (asia-med GmbH & Co. KG, Germany)
Treatment regimen	(a) Number of treatment sessions	Single treatment session
	(b) Frequency and duration of treatment sessions	During the received chemotherapy administration
Other components of treatment	(a) Details of concurrent interventions	No concurrent interventions
	(b) Setting and context of treatment	Outpatient clinic, all treatments in a specific study center will be performed by the same practitioner
Practitioner background	(a) Description of participating acupuncturists	Licensed Traditional Chinese Medicine practitioner with 5 years of studies at the Tianjin University (China) and 3 years of acupuncture treatment experience in a clinical oncological setting
Control or comparator interventions	(a) Rationale for the control or comparator	Non-penetrating sham acupuncture
	(b) Precise description of the control or comparator	Sham points: 1 to 1.5 cun (1 to 1.5 thumb’s width) beside the verum acupuncture points using the dull side of the needle

#### Nonpenetrating sham acupuncture group

Patients of the sham group receive sham acupuncture under the tongue also during their next chemotherapy administration after study inclusion. Treated points will be 1 to 1.5 cun (a cun is defined as the width of the patient’s thumb at the knuckle) beside the verum acupuncture points Jinjin and Yuye using the dull side of the needle [26, 27].

#### Standard medical care group

Patients of the standard medical care group receive evidence-based dietary recommendations for dysgeusia [2, 12] during their first chemotherapy administration after study inclusion. Dietary advice will be provided by the study physician based on a self-help book of the German Cancer Society [28]. This self-help book was also given to the patients to take home.

### Data collection

Baseline data will be assessed before randomization (T_0_) and will include social demographics (age, sex, family status, education, employment), medical data of the patient (tumor type, staging, grading, relapse, menopausal status, chemotherapy type), and ratings for baseline outcome data.

### Primary outcome

The primary outcome is phantogeusia [11, 29, 30], a continuous abnormal taste sensation without an external oral stimulus, as assessed at follow-up (T_2_) by an 11-point NRS.

### Secondary outcomes

Secondary outcomes include phantogeusia, assessed between T_1_ and T_2_ using a daily patient log, as well as parageusia (abnormal taste of food), hypogeusia (reduced taste sensations), hypergeusia (increased taste sensations), xerostomia (dry mouth), stomatitis symptoms, appetite, and functional/social impairment, assessed at T_2_ and between T_1_ and T_2_ using a daily patient log with 11-point NRSs, respectively.

### Safety assessment

All adverse events will be recorded and assessed by the study physician at T_2_. According to good clinical practice guidelines, adverse events are defined as any untoward medical occurrence in the patient administered a medicinal product and that does not necessarily have a causal relationship with this treatment [31]. Thereby, serious adverse events are defined as any untoward medical occurrence that at any dose resulted in death, was life-threatening, required inpatient hospitalization, or resulted in persistent or significant disability/incapacity [31]. All participants will further be asked about any such event during the study period (between T_1_ and T_2_) and at follow-up (T_2_).

### Treatment expectancy and credibility

Treatment expectancy and credibility will be assessed by the Treatment Credibility Scale [32, 33] and the Bang blinding index [34, 35] to examine possible confounding effects due to expectation and conditioning and to control the success of the blinding toward the verum and sham acupuncture. Expectancy will be assessed at baseline (T_0_), and credibility will be assessed directly after the acupuncture treatment (T_1_) and at follow-up (T_2_).

### Sample size calculation

No study has yet investigated the effect of hypoglossal acupuncture for chemotherapy-induced dysgeusia; as such, a sample size estimation on the basis of previous effect sizes was not possible. Therefore, a sample size of *n* = 25 patients per group was considered sufficient to detect an effect of *d* = 0.8, using a two-sided *t* test between independent groups (verum acupuncture versus standard medical care) with an α of 0.05 and a power of 1 − β = 0.8, as calculated using G*Power software (release 3.1.3; Kiel University, Kiel, Germany) [36].

### Statistical analysis

Data will be analyzed using IBM SPSS Statistics for Windows software, release 22.0 (IBM Corp., Armonk, NY, USA). Patients’ characteristics will be presented separately for each group using descriptive statistics, including mean and standard deviation, median and range, frequencies and proportions, based on the data format and distribution.

Data analysis will be conducted according to the intention-to-treat principle; that is, all randomized patients regardless of compliance or withdrawal will be included. Missing values will be multiply imputed by the Markov chain Monte Carlo method [37, 38], yielding a total of 50 complete datasets. Statistical tests will be conducted in a hierarchical order, starting with the comparison of acupuncture vs. usual care, which in case of significant effects will be followed by the comparison of acupuncture vs. sham acupuncture.

The primary outcome will be analyzed as a function of the treatment group (verum acupuncture versus standard medical care), patients’ expectations, and their respective baseline values (linear covariates) using a univariate analysis of covariance. Between-group differences and 95% confidence intervals were estimated using two-sided *t* tests and an alpha level of 5%. If the analysis reveals a statistically significant between-group difference, a second univariate analysis of covariance on the primary outcome will be conducted as a function of the treatment group (verum acupuncture versus sham acupuncture), patients’ expectations, and their respective baseline values (linear covariates). Between-group differences and 95% confidence intervals were estimated using two-sided *t* tests and an alpha level of again 5%. Because of hierarchical testing of the hypotheses, no alpha level adjustment is necessary [39]. The same covariance models will be applied for secondary outcome measures; for those effect measures, confidence intervals and *P* values will be reported descriptively. Moreover, a Kaplan-Meier survival analysis will be used to analyze group differences regarding the time point of symptom relief based on the daily patient log. Data on tolerability and safety will be reported descriptively. No interim analysis will be conducted.

## Discussion

Dysgeusia is often discussed as a less impairing, less significant side effect of chemotherapy. For patients, however, taste distortions during chemotherapy greatly affect appetite and quality of life, with few available effective treatment options [3, 6, 10]. Acupuncture as a relatively simple and short intervention that did not show severe side effects during the application of chemotherapy [17] and preliminary effects in patients with idiopathic dysgeusia [13] appears to be an important subject of research. The present trial was rigorously designed according to the CONSORT and STRICTA recommendations, including a nonpenetrating sham needling control, blinding of outcome assessors and patients, controlling of blinding success and expectation effects, and the use of standardized NRSs [22]. Particularly regarding the design of an ideal sham acupuncture control procedure, researchers broadly discussed how to mimic best the procedure of acupuncture and control for unspecific effects of expectation, conditioning, and therapeutic attention without provoking specific physiological effects [40–42]. It was argued that noninvasive sham acupuncture appears to be a more suitable control condition than the minimally invasive approach. In this study, using a noninvasive sham procedure should be feasible and blindable because participants do not have a visual control.

This trial should therefore generate the first evidence for the efficacy and effectiveness of hypoglossal acupuncture in patients with breast cancer who have acute chemotherapy-induced dysgeusia. Results can provide suitable information for further sham protocols and clinical acupuncture trials.

## Trial status

This randomized clinical trial is currently recruiting participants.

## Data Availability

Not applicable.

## Abbreviations

CONSORT: Consolidated Standards of Reporting Trials
NRS: Numeric rating scale
SPIRIT: Standard Protocol Items: Recommendations for Interventional Trials
STRICTA: Standards for Reporting Interventions in Clinical Trials of Acupuncture
T: Time point
TNM: Tumor, node, metastasis, classification of malignant tumors
